# Cytokines as Prognostic Biomarkers of Epithelial Ovarian Cancer (EOC): A Systematic Review and Meta-Analysis

**DOI:** 10.31557/APJCP.2021.22.2.315

**Published:** 2021-02

**Authors:** Moh Nailul Fahmi, Heru Pradjatmo, Indwiani Astuti, Ricvan Dana Nindrea

**Affiliations:** 1 *Department of Obstetrics and Gynaecology, Faculty of Medicine, Public Health, and Nursing, Universitas Gadjah Mada, Dr Sardjito Hospital, Yogyakarta, Indonesia. *; 2 *Department of Pharmacology and Therapy, Faculty of Medicine, Public Health, and Nursing, Universitas Gadjah Mada, Yogyakarta, Indonesia. *; 3 *Department of Public Health and Community Medicine, Faculty of Medicine, Universitas Andalas, Padang, Indonesia. *

**Keywords:** VEGF, cytokines, prognostic biomarker, epithelial ovarian cancer

## Abstract

**Objectives:**

The value of cytokines as epithelial ovarian cancer (EOC) prognostic factors has been widely investigated. This study aimed to determine the role of single cytokine as a biomarker prognosis in EOC.

**Materials and Methods:**

We conducted a systematic review and meta-analysis of studies reporting cytokine as the prognostic predictor in EOC based on PRISMA guideline. We included English articles investigating associations of preoperative cytokines level in tissue, blood or ascites with overall survival (OS) or disease-free survival (DFS) from PUBMED and EBSCO. Summary hazard ratios (HRs) and confidence intervals (CIs) were calculated.

**Results:**

Fifty studies investigating twenty types of cytokines in tumor tissue, serum, and ascites from 5,376 patients were included. Pre-operative high VEGF level was associated with poor OS (HR 2.28, 95%CI [1.28, 3.28]) and DFS (HR 2.13, 95%CI [1.63, 2.78]) in serum and OS (HR 1.80, 95%CI [1.45, 2.23]) in tissue. IL-6 level in blood was associated with DFS (HR 1.60, 95%CI [1.21, 2.11]). There was no single cytokine which investigated by at least 2 studies reporting hazard ratio in ascites, so we did not conduct the meta-analysis. Other cytokines (serum IL-8; ascites fluid IL-8, IL-10, IFN-γ, TNF-α; and ovarian tissue TGF-α, CSF-1, IL-10 ,TGF-β1, IL-17) associated with the poorer prognosis, could not be pooled due to lack of studies.

**Conclusion:**

Pre-operative VEGF level in serum and tissue specimen seem to be the potential candidate of an unfavorable prognostic biomarker for EOC. The evidence was lacking to support the other cytokines investigated in blood, tissue and ascites as prognostic biomarkers for EOC.

## Introduction

Ovarian cancer known as a ‘silent lady killer’ due to high mortality and lack of early screening resulting in delayed diagnosis. In the United States, the mortality of ovarian cancer was 6.7 per 100,000 in 2015 (Torre et al., 2018). Various strategies in early detection, diagnosis, treatment and prognosis of ovarian cancer have been investigated. Existing treatments of epithelial ovarian cancer (EOC) include surgery and chemotherapy (Ledermann et al., 2013). However, the prognosis of patients who undergo those treatments are poor. The 5-year cause-specific survival of EOC for all stages is 47% (Torre et al., 2018). There are many factors affecting survival in EOC, for instance, cancer stage, age at diagnosis, histopathological type, pathological grade, surgical status, residual disease, performance status, and biological factors such as proteins and gene expressions (Onal et al., 2017). Prognostic factors may help clinicians in deciding on types of surgery and adjuvant therapies according to individual risks. The role of immunology in ovarian cancer has been investigated in many studies. The competence to predict ovarian cancer cells’ behavior in molecular levels either in blood, tissue or ascites would be beneficial for patients and clinicians in the decision-making process.

The tumor micro-environment of ovarian cancer consists of various cells and molecules that promote or suppress tumor cells. In addition to cancer cells, in the microenvironment of ovarian cancer, there are various types of immune and stromal cells, extracellular matrix molecules, cytokines and chemokines (Musrap and Diamandis, 2012). Cytokines are low molecular weight proteins that mediate intracellular communications. Cytokines are produced by tumor cells, immune cells, and stromal cells like fibroblasts and endothelial cells that might be able to regulate proliferation, survival, differentiation, migration and death. The role of cytokines in ovarian cancer pathogenesis has been investigated in many studies. Most cytokines are found in normal ovarian tissue and are also found associated with malignancy in different functions. Cytokines could be anti-tumor cells but on the other hand, they could be tumor promotors. In some studies, several cytokines were found increasing in patients with EOC including Vascular Endothelial Growth Factor (VEGF), Interleukin 6 (IL 6), IL 8, and IL 10. There are many variations in existing studies in terms of the types of cytokines, cut-off level, methods of measurement, and type of specimens used. Many studies regarding the role of cytokines in survival of EOC have been conducted, but there is no systematic review discussing what kind of cytokines in EOCs that affect survival, from what specimens the sample is collected (tissue, blood or ascites) and what are the most widely used methods.

In this study, we conducted a systematic review and meta-analysis of the prognostic roles of cytokines in EOC from published studies. This study was aimed to determine the roles of cytokines in overall survival (OS) and disease-free survival (DFS) of patients with EOC. The secondary aim was to identify the specimens and methodological characteristics used among the studies that may describe variations.

## Materials and Methods

*Search strategy*

**Figure 1.**
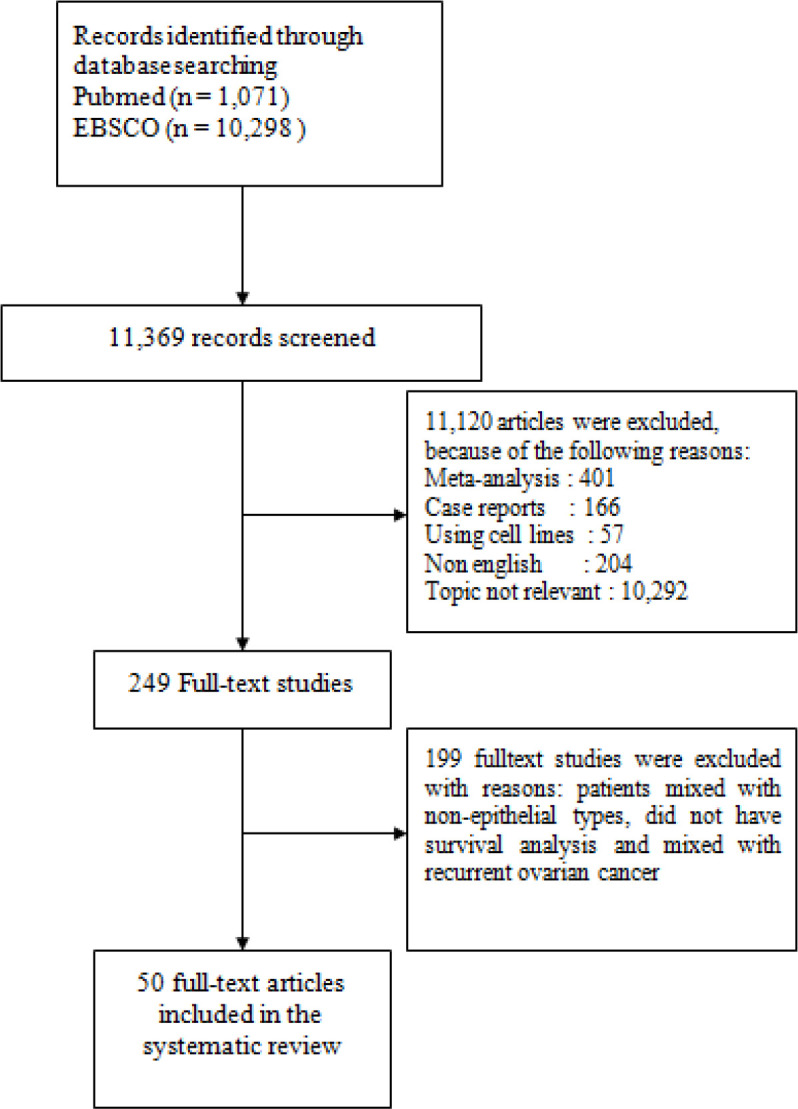
Flow Diagram of Study Selection

This study was conducted based on PRISMA guidelines. We used search terms [(ovary or ovarian) and (cancer or cancers or carcinoma* or neoplasm* or malignant* or tumour or tumor)] or [“ovarian neoplasms” (MESH)], [“cytokines” or “proinflammatory cytokines” or “anti-inflammatory cytokines” or “chemotactic cytokines” or “proangiogenic cytokines”]. We searched literatures from PUBMED and EBSCO from 1988 up to 2018 as shown in Figure 1.

*Selection criteria*

*Types of study*

We included the following characteristics of studies: full-text original articles published in English, prospective or retrospective cohort or case control studies, investigated association individual cytokines in tissue, blood or ascites with OS or DFS of patients with EOC. We excluded the following studies: letters to the editor, reviews or systematic reviews or meta-analysis, case reports or case series, using cell lines, and those that did not report estimation of the effects (P-values, hazard ratio and confidence interval).

*Types of participant*

We included patients who undergone primary surgery for EOC. We excluded patients with history of chemotherapy and previous surgery, and patients with co-existing primary malignancy in other organs.

*Data extraction and study selection*

We independently scanned the titles of articles that we searched to exclude irrelevant studies. We reviewed abstracts of the remaining articles to find the potentially relevant studies and excluded duplicated study. Full-texts of potentially relevant studies were reviewed to be included in this study based on eligibility criteria. We resolved disagreements by discussion and, if required, we involved a third reviewer to make the final decision. The data was extracted from included studies including: first author, year of publication, country, study design, types of cytokines, measurement test methods, sample specimens, sample size, cut-off measurements, methods for determining cut-off, times of follow-up and estimation effect. The endpoints of this study were OS and DFS. 

*Quality assessment*

**Figure 2:**
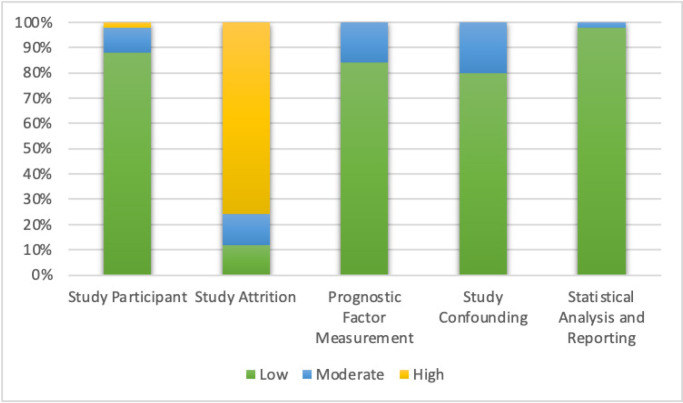
Quality of Included Studies Using QUIPS

Study quality was reviewed by two authors. To assess the quality of included study, we used Quality in Prognosis Study (QUIPS) tools. We placed each included study into ‘low’, ‘moderate’ and ‘high’ risk of bias in six domains (study participant, study attrition, prognostic factor measurement, outcome measurement, study confounding and statistical analysis and report) as shown in Figure 2.

*Statistical analysis*

The STATA software of version 14.2 was performed by clinical epidemiologist and biostatistician in meta-analysis to get the value of pooled hazard ratio of cytokine level in EOC. There were pooled hazard ratio with 95% confidence intervals. Significant heterogeneity was indicated by I_2_>50%. A random effect model was used when significant heterogeneity was observed. Otherwise, a fixed effect model was performed. Publication bias was visually evaluated by using Egger’s and Begg’s tests. 

## Results

A total of 11,369 publications were obtained from PUBMED and EBSCO using our search method. After reviewing the titles and abstracts, we excluded 11,120 citations for several reasons including study design (meta-analysis studies, case reports, and RCTs), non-English languages, and inappropriate topics. From the number of citations, we reviewed the full text and finally included 249 studies that investigated the prognostic role of cytokines as biomarkers for ovarian cancer. After a deep review, 199 full text studies were excluded for some reasons including patients with mixed epithelial and non-epithelial types, did not have survival analysis and investigated mixed with recurrent ovarian cancer. There were 50 studies which matched the inclusion and exclusion criteria of this study (Suppl 1).

Fifty studies analyzed the role of various cytokines as prognostic factors of epithelial ovarian cancer. The studies were conducted in European countries (24 studies), and Asian countries (15 studies). One study was a multicenter study with subjects collected in United States, Japan, and United Kingdom (Matsuo et al., 2020). Fifteen studies investigated serum cytokines’ level (Suppl 2), 28 studies sought for cytokines’ expression in ovarian tissues (Suppl 3), while 9 studies measured cytokines’ level in ascites fluid (Suppl 4).

*Study design*

Fifty studies investigated more than 20 types of cytokines (VEGF, IL-1β, IL-2, IL-4, IL-5, IL-6, IL-7, IL-8, IL-10, IL-12, IL-13, IL-15, IL-17, IL-21, Transforming Growth Factor-alpha (TGF-α), TGF-β1, Colony Stimulating Factor-1 (CSF-1), Interferon-alpha (IFN-α), IFN- γ and Tumor Growth Factor-alpha (TNF-α). All of the studies used cohort design to assess the prognostic values of each of the cytokines, and 16 of them were retrospective study. 

Only five studies focused on advanced stage ovarian cancer (Raspollini et al., 2004; Siddiqui et al., 2011; Lan et al., 2013; Kolomeyevskaya et al., 2015; Lane et al., 2015). We know that some study groups shared the same sample, but it was difficult to determine whether a number of samples were taken from the same sample banks or from the fresh subjects. 

*Sample size*

Sample size was varied from 37 up to 320 participants among studies. The total number of participants in this review were 5,376. Twelve studies did not include control groups. The rest of them involved either normal or benign, or borderline ovarian tumor as control groups.

*Measurement methods*

Immunohistochemistry staining on paraffin-embedded was the most commonly used method in tissue. To evaluate the staining of epithelial cells, the studies used quantitative, qualitative, or semi-quantitative scoring methods. The scoring system was varied among studies.

Nine studies in serum used Enzyme-linked immunosorbent assays (ELISA) as measurement tools. Two studies utilized sandwich enzyme immune-assay technique to quantify serum VEGF-C levels (Cheng et al., 2013; Liang et al., 2013). One study uses western blot method to measure VEGF-A, VEGF-D, and VEGF-C level in serum (Kuerti et al., 2017). Two studies used commercially available multiple panel cytokines assay which was based on sandwich immunoassay to simultaneously investigate various cytokines in a single sample (Lambeck et al., 2007; Aune et al., 2012). Studies measuring cytokine level in ascites commonly utilized ELISA method, while four study used sandwich immunoassay (Liang et al., 2013) and commercially available multiple panel cytokines assay (Matte et al., 2012; Chen et al., 2015; Kolomeyevskaya et al., 2015). 

*Cytokines level in serum and prognosis of EOC*

**Figure 3:**
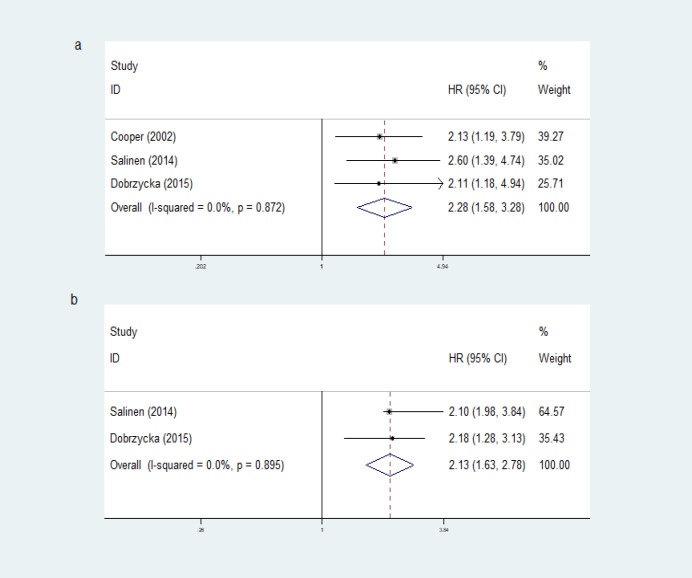
Forest Plots VEGF Level in Serum for OS and DFS. Noted: a, VEGF level in serum for OS; b, VEGF level in serum for DFS

There were two cytokines commonly investigated in serum, VEGF and IL-6. Nine studies reviewed the association between serum VEGF and EOC prognosis. Two studies investigated VEGF-C (Cheng et al., 2013; Liang et al., 2013), one study researched about VEGF-165 (Mahner et al., 2010), an another six studies reviewed VEGF (VEGF-A). All of those study consistently showed that higher levels of VEGF were associated with poor OS, except for one study which stated that preoperative VEGF levels were not associated with OS (Mahner et al., 2010). All of the four studies assessing DFS or recurrence-free survival (RFS) showed that higher VEGF level was associated with poor prognosis. In this meta-analysis, high VEGF level was associated with poor OS (HR 2.28, 95%CI [1.28, 3.28], fixed effect) and DFS (HR 2.13, 95%CI [1.63, 2.78], fixed effect) as shown in Figure 3. Heterogeneity on meta-analysis study of the VEGF level in serum for the survival (p heterogeneity = >0.05; I^2^ = <50%) showed a variation of homogeneous research. There were wide ranges of cut-off value, from 171 pg/mL (Mahner et al., 2010) up to 10.200 pg/mL (Cheng et al., 2013). Most of the cut-off values were defined from median values, and the remaining were based on 95% percentile (Ska et al., 2004), 75% quartile (Chen et al., 1999), and taken from previous studies (Chen et al., 1999; Hefler et al., 2006). 

**Figure 4:**
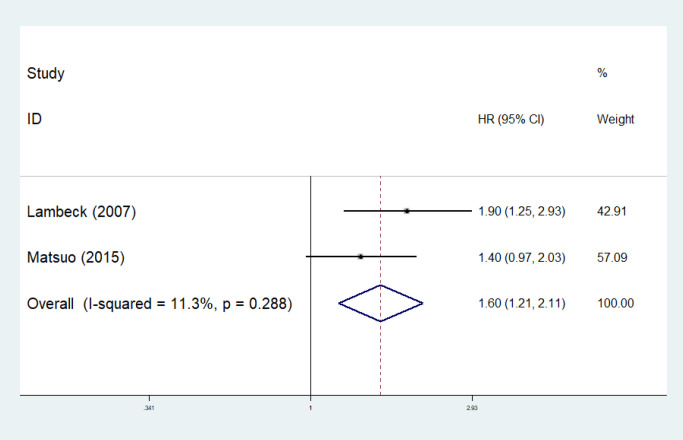
Forest Plots IL-6 Level in Serum for DFS

Five studies investigated blood IL-6 level as a prognostic factor of EOC. There was an association between IL-6 level in blood for DFS (HR 1.60, 95%CI [1.21, 2.11], fixed effect) as shown in Figure 4. Heterogeneity of this study (p heterogeneity = >0.05; I^2 ^= <50%) showed a variation of homogeneous research. Three of those studies (Scambia et al., 1995; Tempfer et al., 1997; Lambeck et al., 2007), showed significant association between high level of IL-6 and poor OS, while one of them (Kumar et al., 2017) showed no significant association. Two studies revealed significant association between higher blood IL-6 with shorter progression and DFS, while one study showed no significant association (Matsuo et al., 2020). Cut-off value was varied from 0.7 pg/mL (Tempfer et al., 1997) to 24 pg/mL (Kumar et al., 2017). One study investigated the association of serum IL-8 level and OS and found that more than 59 pg/mL serum IL-8 level was related to worse OS (Aune et al., 2012). 

*Cytokines expression in tissue and prognosis of EOC*

**Figure 5:**
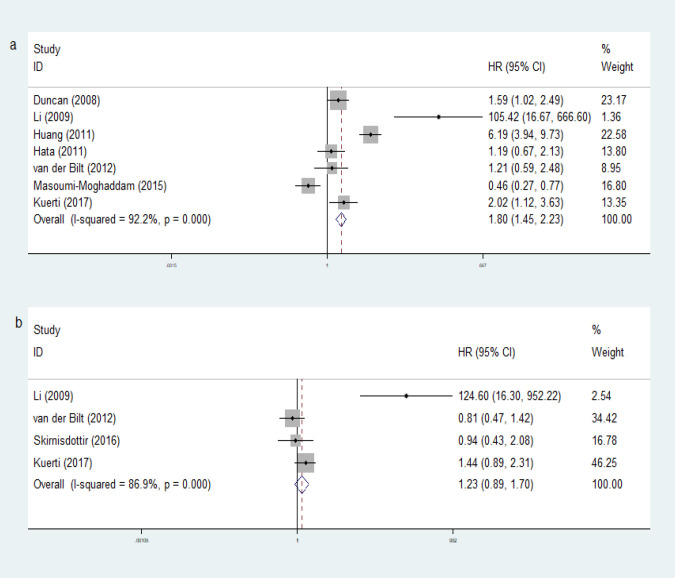
Forest Plots VEGF Level in Cancer Tissue for OS and DFS

Twenty-four studies reviewed VEGF and the isoforms (VEGF-A, VEGF-B, VEGF-C, and VEGF-D). Twenty studies investigated VEGF (VEGF-A), while the rest of them studied VEGF-B (1 study), VEGF-C (4 studies), and VEGF-D (1 study). Nineteen of them analyzed their effect on OS, while 14 of them analyzed the DFS or progression free survival (PFS). As shown in Figure 5, high VEGF level in cancer tissue was significantly associated with poor OS (HR 1.80, 95%CI [1.45, 2.23], random effect), but no significant association with DFS (HR 1.23, 95%CI [0.89, 1.70], random effect). Heterogeneity for this study (p heterogeneity = <0.05; I^2 ^= >50%) showed a variation of heterogeneous research. Eleven studies (58%) found that higher expression of VEGF was correlated with shorter OS (Shen et al., 2000; Kassim et al., 2004; Raspollini et al., 2004; O’Toole et al., 2007; Duncan et al., 2008; Li et al., 2009; Smerdel et al., 2009; Siddiqui et al., 2011; Williams et al., 2012; Sui, 2012; Kuerti et al., 2017). One study found that expression of VEGF-C and VEGF-R2 were associated with poor OS while VEGF-A and VEGF-R3 expressions were not significantly associated (Nishida et al., 2004). The study performed by Yokoyama found that positive VEGF-D was associated with reduced 10-years survival rates, while positive VEGF-C and VEGFR-3 were not significantly associated with 10-years survival (Yokoyama et al., 2003). Another five studies showed no significant effect of VEGF expression on OS. In contrast, only two studies showed that higher VEGF expression were associated with prolonged OS (Masoumi-moghaddam et al., 2015; Liu et al., 2012). Fourteen studies investigated PFS of the patients with VEGF expression of ovarian tissue. Six of them found that positive VEGF expression significantly reduced PFS (Zhang et al., 2003; Brustmann, 2004; Nishida et al., 2004; Raspollini et al., 2004; O’Toole et al., 2007; Li et al., 2009). Two studies showed improvement of PFS in tissue with positive VEGF expression (Ogawa et al., 2002; Engels et al., 2009). Another six studies showed no significant association between VEGF expression with prognosis related to PFS.

Another five studies investigating six different cytokines found expression of some cytokines were correlated with prognosis. Expressions of IL-17 and TGF-β were correlated with better RFS (D’Antonio et al., 2002; Droeser et al., 2013). Expressions of TGF-β1 and CSF-1 were associated with poor OS and DFS, respectively (Chambers, 1997; Liu et al., 2012). Expressions of IL-10 and IL-6 in ovarian tissue were not significantly correlated to OS (Liu et al., 2012; Masoumi-moghaddam et al., 2015).

*Cytokines level in ascites and prognosis of EOC*

Fourteen studies assessed the association between cytokines level in ascites and prognosis of EOC. Four of them were studies of IL-6, three studies of IL-10, three studies of VEGF, one study of IFN-γ, two studies of TNF-α, and one study of IL-8.

We did not conduct a meta-analysis in ascites due to there was no cytokine that investigated by at least 2 studies reporting hazard ratio. 

Two studies investigating IL-6 showed shortened PFS in patients with higher IL-6 levels (Lane et al., 2015; Dalal et al., 2018). Kolomeyevskaya et al. found that Il-6 level in ascites level was not independently correlated to PFS and OS (Kolomeyevskaya et al., 2015). Meanwhile, if high IL-6 level (>4741.6 pg/ml) was combined with higher TNF-α (>38.5 pg/m), they give shorter PFS (median value 6.2 months vs 1.2 months, log rank p = 0.00015). 

Only one study investigating IL-10 found that higher level of ascites IL-10 serum was associated with poor PFS, while another two studies showed no significant correlation. Two studies reviewed the correlation of VEGF (VEGF-A) and prognosis, while one study reviewed VEGF-C. VEGF level in ascites fluid consistently correlated to poor prognosis. One of three studies reviewing ascites’ VEGF found that increasing level of VEGF-C was associated with shorter OS (Liang et al., 2013), while the other study showed that high VEGF-A level was associated with shorter PFS (Dalal et al., 2018). Another study showed that VEGF level higher than 2575 pg/mL was associated with significantly shorter OS and PFS (Rudlowski, 2006). Some studies analyzed the correlation between VEGF expression and tumor stage, which showed that positive or high VEGF expression was correlated with advanced stage tumor, however, this correlation was not consistently significant.

Study about IFN-γ in ascites found that higher IFN-γ was correlated with reduction of both OS and PFS (Chen et al., 2013). The same correlation was found in IL-8 and TNF-α (Kolomeyevskaya et al., 2015).

*Publication Bias*

**Figure 6.**
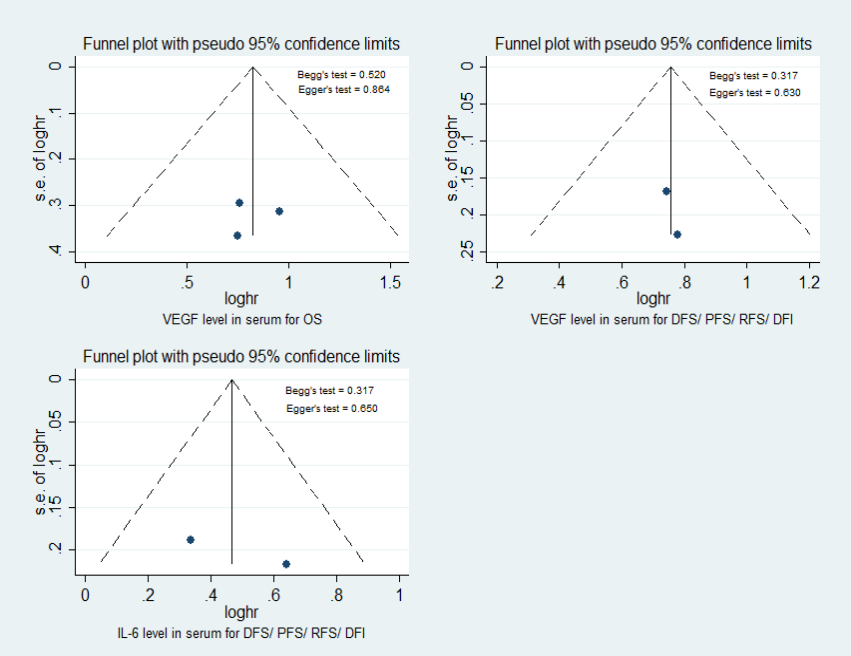
Funnel Plot to Examine Publication Bias for Cytokine Level in Serum for OS and DFS

**Figure 7:**
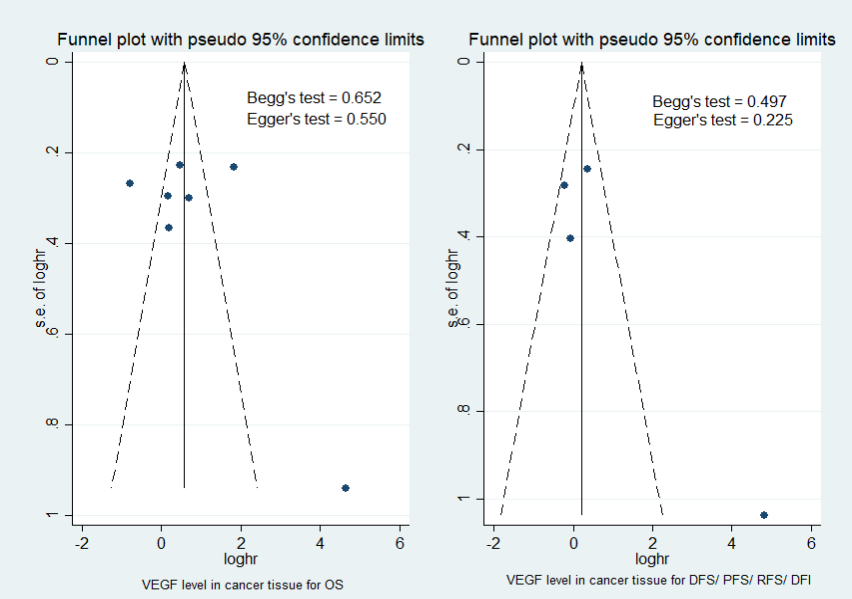
Funnel Plot to Examine Publication Bias for Cytokine Level in Cancer Tissue for OS and DFS

As shown in Figure 6, the Egger’s and Begg’s test were used to evaluate potential bias. No publication bias was confirmed to exist in serum for VEGF and IL-6 level (p>0.05). In cancer tissue, there was not significant publication bias for studies on cytokine level for OS and DFS (p>0.05) as shown in Figure 7. 

## Discussion

In this study, we conducted a systematic review and meta-analysis of the cytokines as prognostic biomarkers in EOC. We found that significant association between high VEGF level in serum with poor OS and DFS. In tissue high VEGF level was correlated with OS but not in DFS. This finding indicates that VEGF has a role in carcinogenesis and progression of disease in EOC. High VEGF level in serum could be a potential biomarker of prognosis. 

VEGF family consists of seven isoforms; VEGF-A, VEGF-B, VEGF-C, VEGF-D, VEGF-E, VEGF-F, and Placental Growth Factor (PIGF) (Parveen et al., 2019). The main isoform which is important for angiogenesis is VEGF-A and is simply referred to as VEGF (Gavalas et al., 2013).

Preclinical experiments have shown that overexpression of VEGF can convert normal ovarian epithelium into neoplastic tissue which produces ascites. VEGF expression in omental metastases, cysts fluid, ascitic fluids and blood of EOC patients indicate the role of VEGF in the process of carcinogenesis. In addition, overexpression of VEGF receptors and co-receptors has been found in ovarian cancer (Masoumi Moghaddam et al., 2012). 

VEGF and Platelet Derived Growth Factor (PDGF) are secreted by cancer cells. These factors activate endothelial cells, thus causing the formation of new blood vessels to initiate angiogenesis (Gavalas et al., 2013). The newly formed vessels infiltrate tumor mass in the local tumor microenvironment and promote tumor mass expansion, and subsequent hematogenous metastasis spread, thereby contributing to cancer progression (Masoumi Moghaddam et al., 2012). VEGF ligands bind various tyrosine kinases and non-tyrosine kinase receptors involved in cancer development (Parveen et al., 2019). Binding of the ligand to the receptor induces receptor dimerization that leads to the initiation of intracellular signaling (Gavalas et al., 2013).

VEGF derived from ovarian cancer cells increases the regulation of angiopoietin 2 in host endothelial cells and induces paracrine remodeling of the host blood vessels to support angiogenesis during tumor growth. VEGF secretion can activate the Akt1 and Akt3 pathways, two downstream effectors of PI3K signaling pathways. Akt1 and Akt3 have their important role in ovarian carcinogenesis. In ovarian cancer there is an increase in tumor growth through the autocrine loop mechanism by the expression of VEGFR-2 in cancer cells along with VEGF (Masoumi Moghaddam et al., 2012).

VEGF has an important role in the aggressiveness and metastasis by directly stimulating proliferation, survival, and/or migration of tumor cells, producing blood vessels, forming ascitic fluid and producing matrix which are important for tumor blood supply. The role of VEGF-VEGFR in invasion and migration of ovarian cancer is proven in vitro through the secretion and activation of Matrix Metalloproteinase-2 (MMP-2), MMP-7, MMP-9 and urokinase type plasminogen activators. VEGF contributes to intraperitoneal spread of ovarian cancer by promoting neovascularization and increasing vascular permeability leading to subsequent growth of intraperitoneal tumors, development of peritoneal carcinomatosis, and formation of malignant ascites. The role of VEGF in peritoneal metastasis of ovarian cancer has been explored by different researchers. Increased ascites accumulation is not only caused by tumor growth through stimulation of angiogenesis but also directly through its ability to increase permeability of peritoneal blood vessels. Intraperitoneal carcinomatosis has components that depend on the presence of the angiogenesis process needed by solid tumors for neovascularization to grow larger and have components that do not depend on angiogenesis such as thin layers of tumors and some small solid tumors that survive through passive diffusion of nutrients from the host blood vessels and fluid in the peritoneum around it (Masoumi Moghaddam et al., 2012).

The wide range of cut-off values of VEGF levels among the studies can be caused by various factors, such as host factors (including genetic influence, ethnic, comorbidities, environment, lifestyle, and habits), samples collection, methods and duration of storage, and measurement tools. 

In tissue specimens, eleven studies found that high VEGF expressions were associated with poor OS. Expression of VEGF in cancer tissue was not consistently correlated to PFS. While 22 studies revealed tissue VEGF expression as a poor prognostic factor for survival, two studies found the contrasting results. Ogawa showed correlation between higher VEGF expression and improvement of PFS, but it was only significant for early stage cancer. The author explained that heterogeneity in most of the slides was the reason for this paradoxical result (Ogawa et al., 2002). Engel said that positive correlation between positive VEGF and prolonged PFS was due to increased vessel permeability effect of VEGF, thus improving drug delivery to tumor tissue (Engels et al., 2009). However, there is no scientific evidence of such mechanisms.

Although there were three studies investigating the significant correlation between high VEGF level and poor prognosis, but could not be analyzed in meta-analysis because only one study reporting the hazard ratio. Some studies in ascites specimen analyzed the correlation between VEGF expression and tumor stage, which showed that positive or high VEGF expression was correlated with advanced stage tumor, however, this correlation was not consistently significant. 

In this systematic review, the other cytokine that was most commonly studied, in addition to VEGF, is IL-6. In meta-analysis, two studies showed that serum IL-6 were associated with DFS. More studies with larger sample size and multivariate analysis are required to determine the role of IL-6 in EOC prognosis.

We found that some other cytokines such as serum IL-8, ascites fluid IFN-γ and TNF-α; and ovarian tissue TGF-α, CSF-1, and TGF-β1 were associated with poorer prognosis of EOC, but only seven studies investigated those correlations. Therefore, we can conclude that, the evidence was limited and not sufficient to determine the prognostic value of those biomarkers.

Based on this systematic review and meta-analysis, we conclude that VEGF level could be potential candidate biomarkers of OS and DFS in serum and OS in tissue. We recommend that a multicenter, high quality study of serum VEGF with larger sample sizes should be conducted. It should involve a uniform, standardized method of cytokine measurement and include adequate follow-up duration to determine the cut-off value of VEGF to differentiate EOC patients with good or bad prognosis. Another study is expected to find out whether serum VEGF is useful in chemotherapy response follow-up, like the widely-used Ca-125, and in measuring patient response to targeted therapy such as bevacizumab.

In conclusion, pre-operative serum VEGF level in serum and tissue specimen seem to be a potential candidate of an unfavorable prognostic biomarker for epithelial ovarian cancer. The evidence was lacking to support the other cytokines investigated in blood, tissue and ascites as prognostic biomarkers for epithelial ovarian cancer. 
